# Global status and trends of proteomics in obesity: a bibliometric analysis and knowledge graph study

**DOI:** 10.1186/s41182-025-00785-7

**Published:** 2025-08-26

**Authors:** Xiaoying Wu, Yongming Chen, Tong Wu, Qi Pan, Lixin Guo

**Affiliations:** 1 Department of Endocrinology, Beijing Hospital, Peking University Fifth School of Clinical Medicine, National Center of Gerontology, Beijing, China; 2 Department of Urology, Beijing Hospital, National Center of Gerontology, Institute of Geriatric Medicine, Chinese Academy of Medical Sciences, Beijing, China; 3 Department of Orthopedics, Peking University Third Hospital, Beijing, China

**Keywords:** Proteomics, Obesity, Bibliometrics, CiteSpace, VOS viewers

## Abstract

**Aims:**

Proteomics plays an essential role in uncovering the molecular mechanisms of obesity. This study aimed to map global research trends, identify major contributors, and examine evolving themes in this field over the past two decades.

**Methods:**

A bibliometric analysis was performed using the Web of Science Core Collection (WoSCC) from 1999 to February 2025. Only English-language original articles and reviews were included. CiteSpace and VOSviewer were used to analyze publication volume, collaboration networks, core journals, citation patterns, and keyword co-occurrence.

**Results:**

A total of 1688 publications were analyzed. Research output increased steadily and peaked in 2022. The United States led in publication count and citations, followed by China and Germany. Keyword analysis revealed a clear thematic shift: early studies focused on adipose biology and hormonal regulation, while more recent work highlights topics such as gut microbiota, lipid metabolism, type 2 diabetes, and PCOS. These shifts reflect an increased focus on systemic mechanisms and a stronger link to clinical needs such as diagnosis and personalized treatment.

**Conclusion:**

Proteomics research in obesity has grown in both scale and complexity, with expanding global collaboration and evolving scientific priorities. This study provides a comprehensive overview of the field’s development and offers direction for future translational and interdisciplinary efforts.

## Key Points

- Research on proteomics in obesity has grown rapidly in the past 20 years, with a noticeable surge after 2010.
- The U.S., China, and Germany have made major contributions, and recent studies increasingly focus on gut microbiota, lipid metabolism, and related disorders.
- This bibliometric analysis offers a clear picture of global progress and points to key areas for future research and collaboration.

## Background

The global rate of obesity has increased sharply in recent decades. This trend is mainly due to higher energy intake and lower physical activity. These changes are closely linked to socioeconomic development. The World Obesity Atlas 2024, released by the World Obesity Federation, reports that by 2035, around 54% of adults worldwide will have obesity. The total number is expected to reach 1.53 billion. This presents a major challenge to public health and healthcare systems [1]. In the United States, about 172 million adults aged 25 years or older were overweight or obese in 2021. This number is expected to increase to 213 million by 2050. Among them, 146 million individuals are projected to have obesity [2]. Obesity is recognized as a complex and chronic metabolic disease, primarily characterized by abnormal fat accumulation and adipose tissue dysfunction. It is a major risk factor for numerous adverse health outcomes, including type 2 diabetes, hypertension, cardiovascular disease, cancer, and non-alcoholic fatty liver disease [3]. A J-shaped relationship has been observed between body mass index (BMI) and all-cause mortality. Higher BMI is linked to shorter life expectancy [4]. Although progress has been made in understanding and treating obesity, it remains difficult to assess individual risk and predict outcomes. More research is needed to clarify the biological mechanisms that drive obesity. It is also important to identify reliable biomarkers. These efforts will support more accurate diagnosis, guide personalized treatment, and improve clinical care [5].

To meet these challenges, the researchers are increasingly turning to molecular profiling technologies that can offer deeper insights into disease biology. Recent progress in molecular profiling technologies has offered new tools to study the biological basis of human diseases, including obesity. Among various omics approaches, proteomics has emerged as particularly valuable. It allows researchers to identify disease-related proteins and measure changes in protein levels during disease development. This helps in early detection, treatment monitoring, and target discovery for precision medicine [6]. Unlike genomics and transcriptomics, proteomics can detect post-translational modifications and protein–protein interactions [7]. These features have made proteomics central to biomarker discovery and precision medicine in obesity-related research.

Although studies on proteomics in obesity have grown rapidly in recent decades, there remains a lack of systematic summaries that reflect how the field has developed and shifted over time. Bibliometric analysis provides a practical means to explore research patterns, examine academic influence, and track the evolution of key themes. In this study, we applied bibliometric analysis to explore the global development of proteomics research in the context of obesity. Rather than merely reporting publication counts or collaboration patterns, we sought to uncover emerging research themes and highlight areas that remain underexplored. Drawing on data from the past 26 years, our analysis covers national and institutional contributions, funding sources, influential researchers, core journals, citation networks, and the evolution of key terms. By providing a comprehensive overview of these dimensions, we aim to offer a valuable resource for scholars in the field and to inform future research strategies.

## Methods

### Search approach

**Fig. 1:**
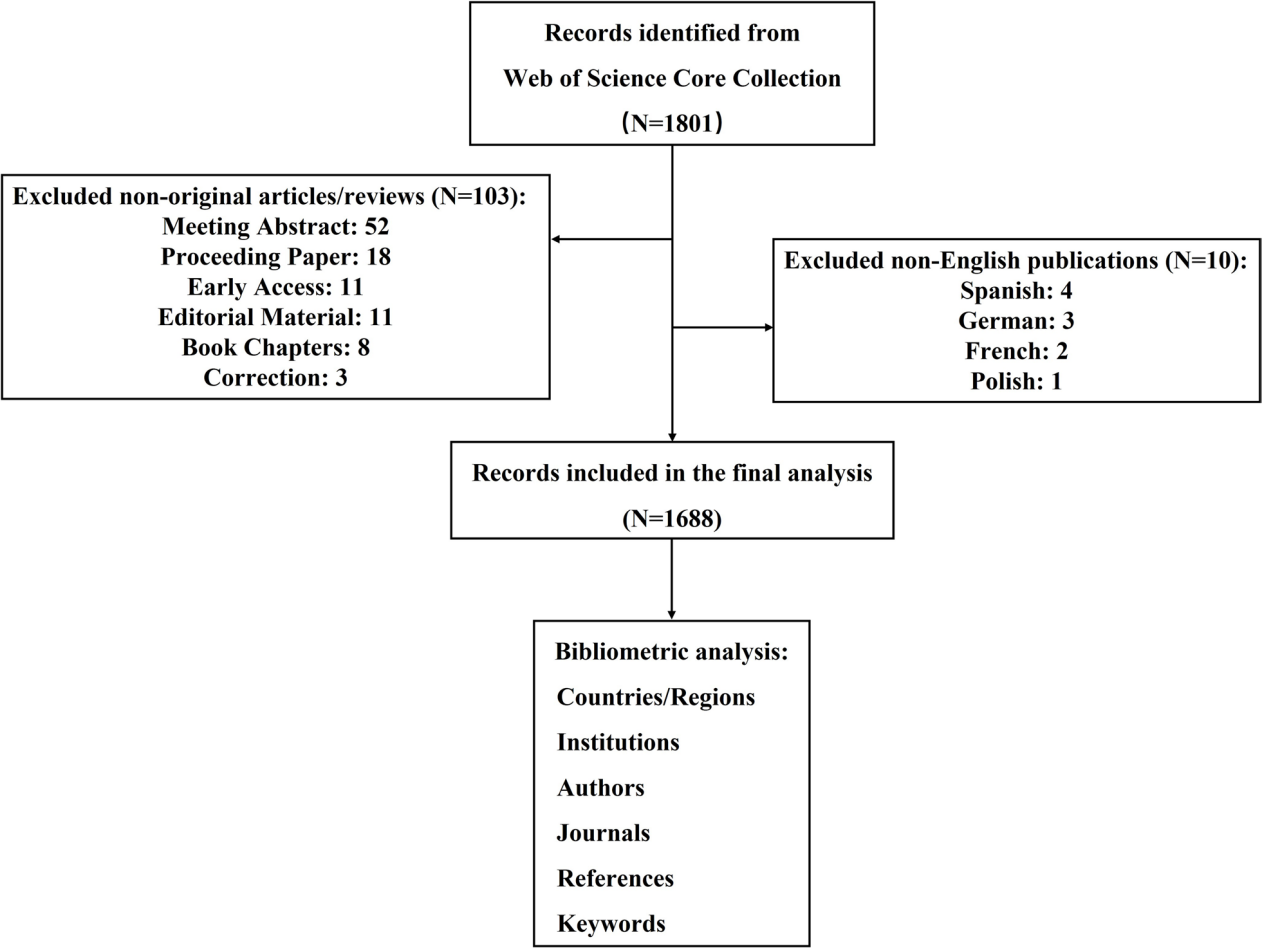
Flowchart of step-by-step literature screening process. A total of 1801 records were identified from the Web of Science Core Collection. After removing non-original articles/reviews and non-English publications, 1688 records were included for bibliometric analysis

In our study, we conducted a bibliometric analysis using the Science Citation Index-Expanded database of WoSCC. Literature retrieval was conducted using the following search logic: TS = (metaproteome OR metaproteomics OR proteome OR proteomics OR metaproteogenomic) AND TS = (obese OR obesity OR obesities OR overweight OR “over weight” OR adiposity). The search was limited to publications from January 1, 1999, to February 12, 2025. The results were exported in both plain text and tab-delimited formats, including full records and reference details. Only English-language original research articles and reviews were included. Initially, 1801 publications were identified; however, 103 were excluded for not meeting the criteria of original articles or reviews, and 10 were removed due to language restrictions (Fig. 1).

### Data analysis

Prior to analysis, we manually standardized author names, affiliations, and keywords to correct for spelling and formatting inconsistencies. The duplicate entries were removed or merged as needed to improve the accuracy of authorship and collaboration data. Two researchers independently downloaded and processed the records using the same retrieval strategy and export settings. Each conducted visualization and mapping analyses with VOSviewer and CiteSpace separately. Their outputs were then compared to check for consistency in clustering and network patterns. Any differences were discussed and resolved before proceeding with the final analysis. The bibliometric information required from the literature, including author names, journal titles, affiliations, countries/regions, publication years, citation counts, reference records, and keywords, was retrieved in plain text and tab-separated formats ("Full Records and Cited References"). These data were then analyzed using VOSviewer (version 1.6.20) and CiteSpace (version 6.3.R1) for country and institutional analysis, journal and co-citation analysis, author and co-cited author analysis, and keyword co-occurrence analysis [8]. By integrating these knowledge mapping and visualization analyses, current research hotspots and trends can be identified.

## Results

### Worldwide trend in publication numbers and citations

**Fig. 2:**
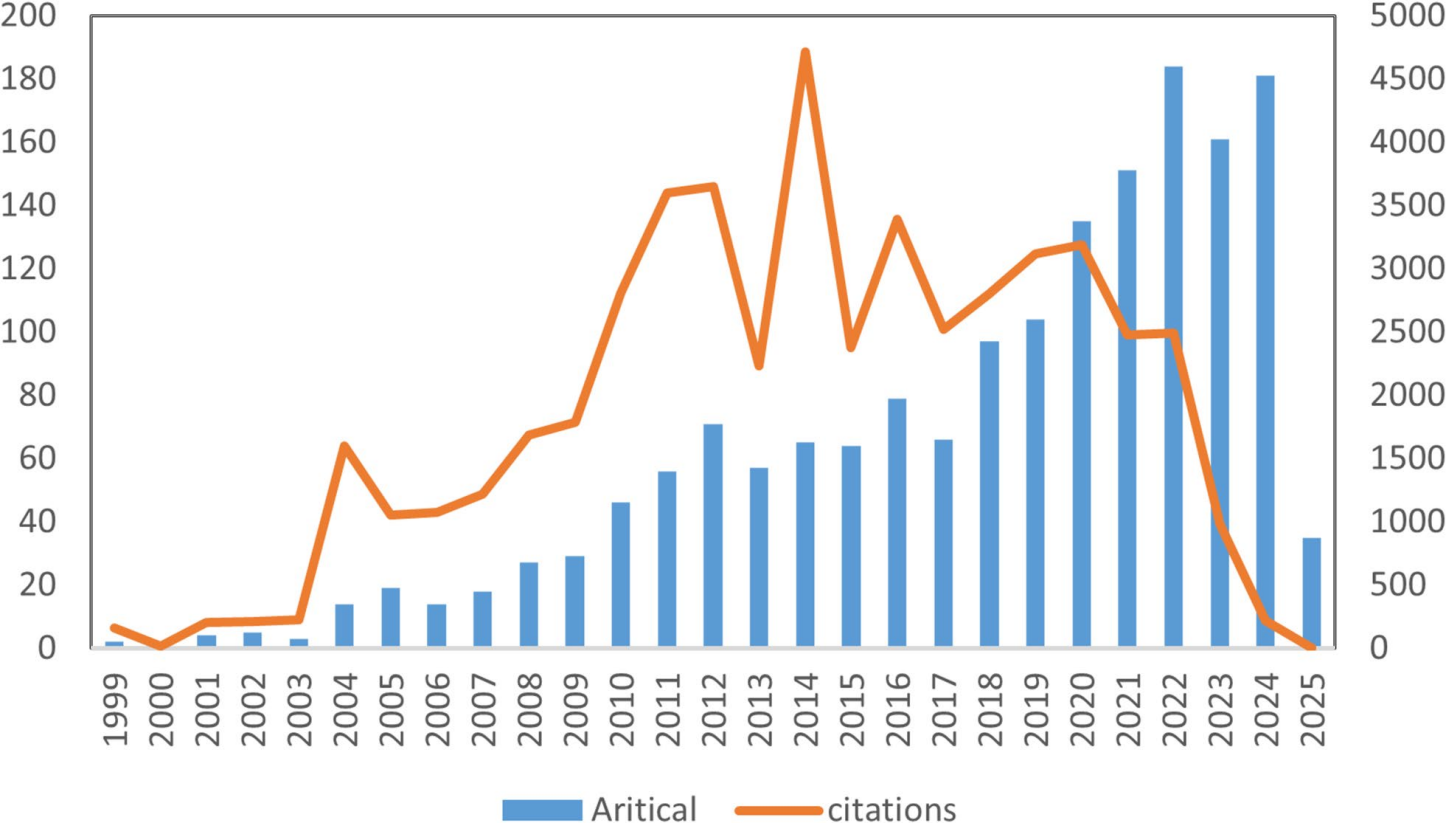
The number of annual publications and citation trend

As shown in Fig. 2, the annual publication output on proteomics in obesity research from 1999 to 2025 exhibited an overall upward trend with slight fluctuations. The highest number of studies was observed in 2022 (*n* = 184), while the lowest was in 1999 (*n* = 2). Between 2004 and 2014, the annual publication output ranged from 14 to 71 articles on average. However, a noticeable surge in the number of publications occurred in the last decade, particularly from 2018 (*n* = 97) to 2022 (*n* = 184). As of the search date, the total citations for all articles amounted to 49,801. From 2010 to 2022, the annual citation count consistently exceeded 3000, highlighting sustained academic attention in this area (Fig. 2).

### Distribution and global contribution of countries/regions, institutions

**Fig. 3:**
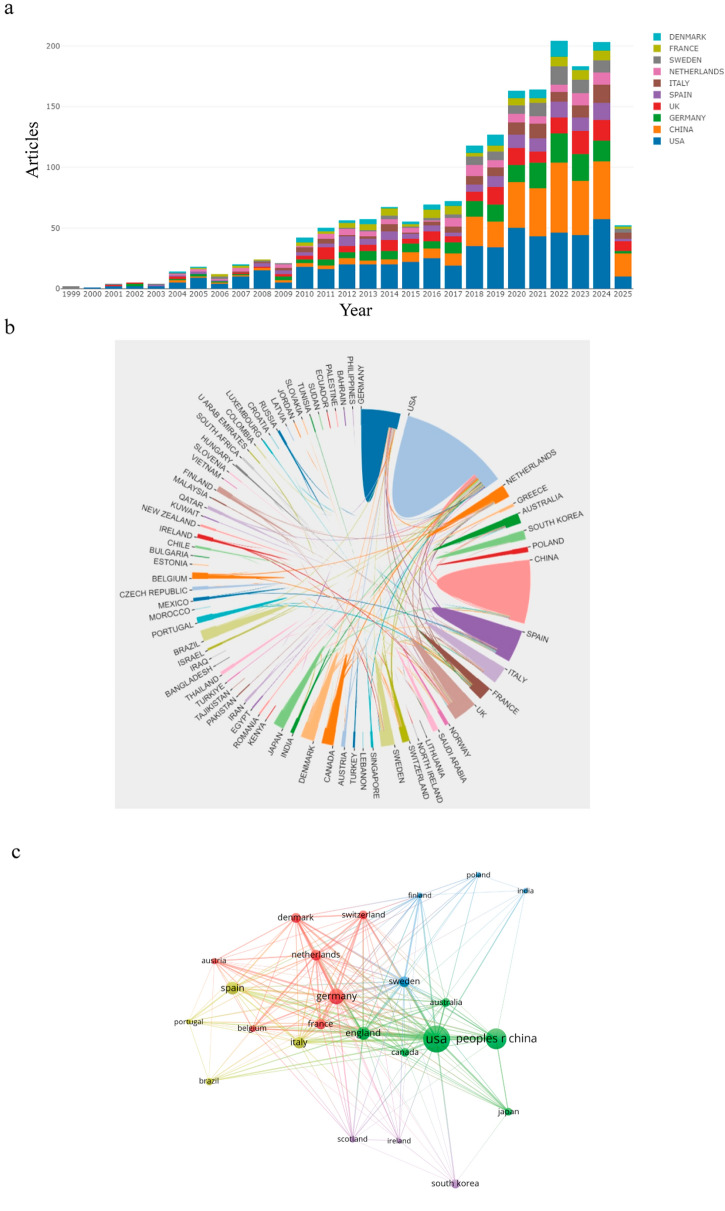
Distribution of published articles across countries and their correlation network. **a** The growth trends of the top 10 countries in field of proteomics in obesity from 1999 to 2025. **b** The international network collaboration among the countries/regions, generated using an online platform for bibliometric analysis. **c** Correlations among countries/regions with over 25 publications, visualized using VOSviewer. Line thickness represents citation strength

Building upon the rising publication trend, we next examined the geographical distribution of research output. Table 1 summarizes the top ten most productive countries/regions in proteomics-related obesity research. As shown in Fig. 3a, the United States led with 534 publications, followed by China (*n* = 322) and Germany (*n* = 187). The U.S. also had the highest H-index (*h* = 72), indicating both volume and impact, with Germany ranking second (*h* = 42). These findings underscore the dominant role of the U.S. in shaping the field. The international collaboration network, constructed using VOSviewer and an online bibliometric platform, included 24 countries with at least 25 publications (Fig. 3b, c). Line thickness reflects the Total Link Strength (TLS), representing collaboration intensity. The U.S. demonstrated extensive partnerships, particularly with China, which emerged as its closest collaborator. In contrast, inter-country collaborations beyond these key players appeared limited, suggesting opportunities for broader global cooperation.

**Table 1:** Leading 10 countries or regions in publication volume related to proteomics and obesity

Rank	Country	Documents	Percentage	TC	AAC	H-index
1	USA	534	31.64	23441	43.9	72
2	China	322	19.08	4795	14.89	35
3	Germany	187	11.08	6181	33.05	42
4	England	136	8.06	5419	39.85	36
5	Spain	126	7.46	3065	24.33	29
6	Italy	104	6.16	2965	28.51	28
7	Netherlands	94	5.57	4036	42.94	30
8	Sweden	92	5.45	2842	30.89	26
9	France	86	5.09	3575	41.57	28
10	Denmark	79	4.68	3450	43.67	31

*TC* Total citations, *AAC* Average article citations, *USA* United States of America

**Fig. 4:**
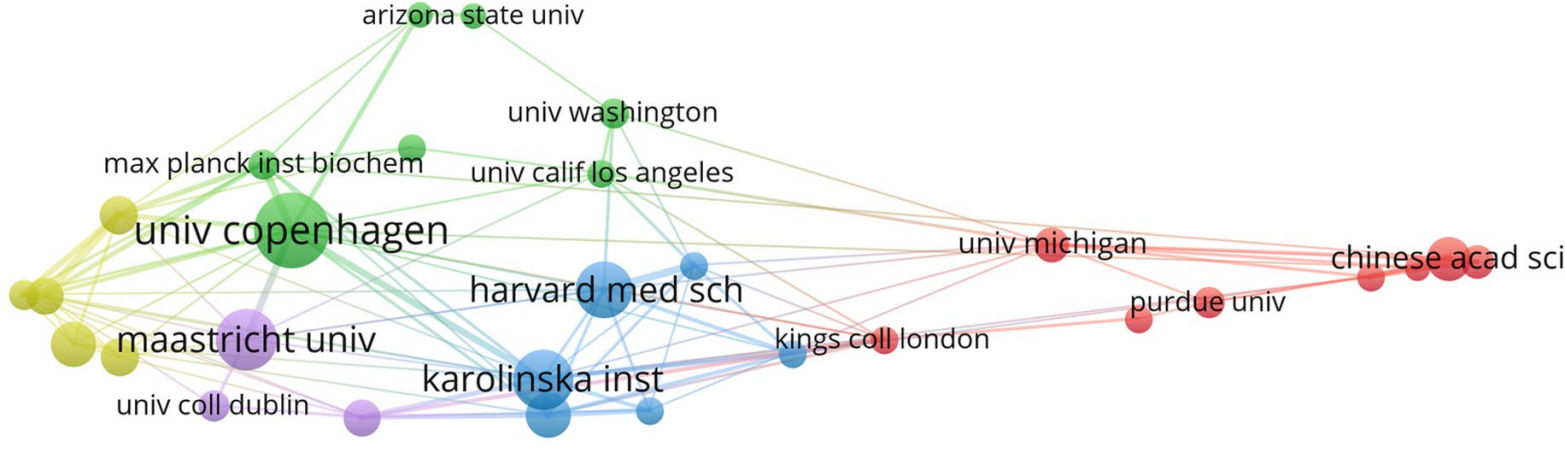
The co-authorship network map of institutions

Turning to the institutional level, Table 2 lists the ten most prolific institutions. Scandinavian and German institutions were prominently represented. The University of Copenhagen ranked first (*n* = 43), followed by Maastricht University (*n* = 35), Karolinska Institute (*n* = 34), and Harvard Medical School (*n* = 32). The institutional collaboration network (Fig. 4) shows node size proportional to publication count, while link thickness represents TLS. The University of Copenhagen (TLS = 46), Karolinska Institute (TLS = 44), and Uppsala University (TLS = 34) were the most collaborative institutions in the network.

**Table 2:** Ten institutions with the highest output in proteomics-obesity studies

Rank	Institutions	Countries/Regions	Articles	Citations	TLS
1	University of Copenhagen	Denmark	43	1495	46
2	Maastricht University	Netherlands	35	1413	15
3	Karolinska Institute	Sweden	34	818	44
4	Harvard Medical School	United States	32	787	26
5	Daegu University	South Korea	29	697	0
6	Uppsala University	Sweden	26	880	34
7	University of Leipzig	Germany	26	694	8
8	Chinese Academy of Sciences	China	25	797	24
9	German Center for Diabetes Research (Deutsches Zentrum für Diabetesforschung, DZD)	Germany	22	581	32
10	Institute of Health Carlos III (Instituto de Salud Carlos III)	Spain	22	378	6

### Contributions of authors and co-cited authors

To further explore individual-level academic influence, we analyzed the productivity and citation impact of researchers in the field. A total of 11,603 authors and 60,229 co-cited authors were identified. Table 3 lists the top ten most productive and most frequently cited contributors in the field of proteomics and obesity. Among them, five authors published more than ten papers, with Yun Jong Won leading in both publication count (*n* = 29) and citations (*n* = 533), and an H-index of 17. Other prolific authors included Choi Jung Won (*n* = 19), von Bergen Martin (*n* = 13), Oh Tae-Seok (*n* = 11), and Chen Shu-Chen (*n* = 11).

**Table 3:** Most active authors and most frequently co-cited researchers in the field

Author	Country	Documents	Citation	AAC	H-Index	Co-cited author	Country	TC
Yun, Jong Won	South Korea	29	553	24.03	17	Cox, J	Germany	697
Choi, Jung Won	South Korea	19	395	27.05	14	Hotamisligil, G. S	USA	514
von Bergen, Martin	Germany	13	721	57.62	12	Huang, D. W	USA	749
Oh, Tae-Seok	South Korea	11	172	23	10	Wisniewski, J. R	Germany	253
Chen, Shu-Chen	China	11	53	6	5	Turnbaugh, P. J	USA	66
Saris, Wim H. M	Netherlands	10	379	42.2	10	Blüher, M	Germany	422
Choi, Duk Kwon	South Korea	10	164	24.3	9	Szklarczyk, D	Switzerland	243
Mariman, Edwin C. M	Netherlands	10	521	54.4	8	Zhang, Y	USA	544
Hojlund, Kurt	Denmark	10	520	62.2	7	Shevchenko, A	Germany	622
Lind, Lars	Sweden	10	59	5.9	4	Rosen, E. D	USA	59

**Fig. 5:**
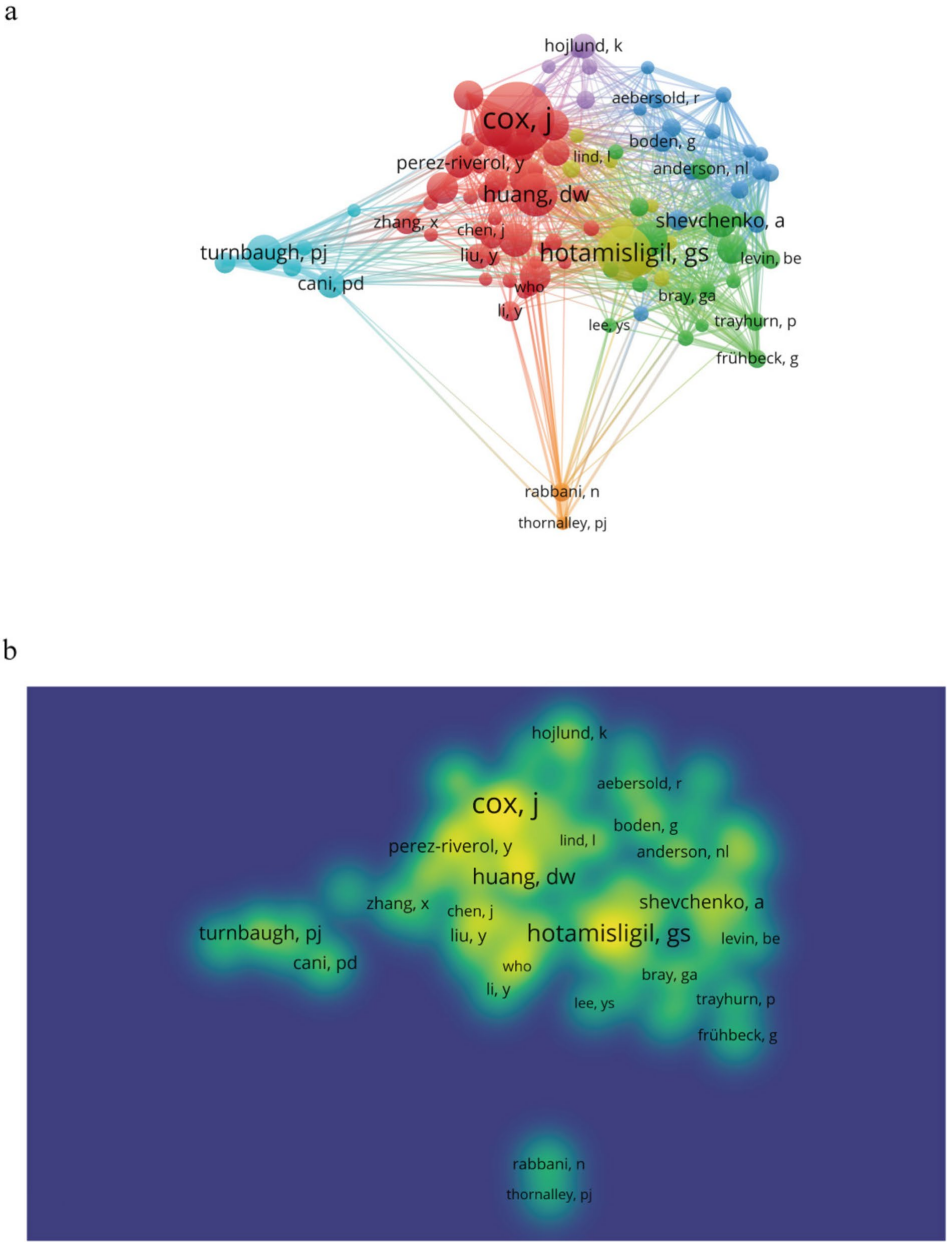
The network (**a**) and density (**b**) visualization of co-cited authors

In addition to authorship output, co-citation analysis was used to assess scholarly influence within the intellectual network. The top ten co-cited authors were cited more than 70 times, with Cox J. ranking first (*n* = 167), followed by Hotamisligil G. S. (*n* = 124) and Huang D. W. (*n* = 99). Based on a threshold of ≥ 30 co-citations, 103 authors were included in the co-citation network analysis, providing insight into influential research collaborations and intellectual structure within the field (Fig. 5a, b).

### Analysis of journals

To understand the publication outlets shaping the field, we identified the top 15 journals publishing articles on proteomics in obesity research. *Proteomics* and *International Journal of Molecular Sciences* were the most productive, with 50 and 48 publications, respectively. Among these journals, Nature Communications had the highest impact factor, while *Biochemical and Biophysical Research Communications* had the lowest. *Molecular & Cellular Proteomics* received the most citations (*n* = 2060), highlighting its influence in the field. Of the 15 journals, 37% were classified as JCR Q1, and 63% had an impact factor above 4.0, suggesting strong journal quality overall (Table 4).

**Table 4:** Fifteen journals publishing the largest number of articles on proteomics and obesity

Rank	Journal	Country	IF (2023)	JCR (2023)	Articles	Citations	ACC	H-Index
1	Proteomics	Germany	3.7	Q2	50	1253	27.78	24
2	International Journal of Molecular Sciences	Switzerland	4.6	Q1	48	497	10.52	15
3	Journal of Proteome Research	United States	4.0	Q2	45	1339	31.87	22
4	PLOS ONE	United States	3.7	Q2	44	1219	28.14	20
5	Scientific Reports	United Kingdom	4.4	Q2	43	733	17.23	18
6	Journal of Proteomics	Netherlands	4.3	Q2	40	731	18.5	17
7	Nutrients	Switzerland	4.2	Q1	35	481	13.8	12
8	Molecular Cellular Proteomics	United States	5.4	Q1	28	2060	79.04	22
9	Frontiers in Endocrinology	Switzerland	3.9	Q2	25	290	11.8	9
10	Obesity	United States	4.1	Q2	21	422	20.43	10
11	Diabetes	United States	9.2	Q1	16	753	50.44	10
12	American Journal of Physiology: Endocrinology and Metabolism	United States	4.0	Q2	15	182	12.13	8
13	Nature Communications	United Kingdom	14.9	Q1	15	646	43.47	9
14	Biochemical and Biophysical Research Communications	United States	3.1	Q3	13	189	14.54	8
15	Cells	Switzerland	5.7	Q1	13	151	11.62	8
16	Frontiers in Physiology	Switzerland	4.1	Q2	13	155	12	8
17	Journal of Lipid Research	United States	4.4	Q2	13	718	55.38	9
18	Molecular Metabolism	Germany	7.9	Q1	13	324	25.08	8
19	Proteomics: Clinical Applications	Germany	3.8	Q2	13	197	15.38	10

### Analysis of co-cited references and citation bursts

To gain insight into how key ideas and research themes have developed, we analyzed co-citation relationships and tracked citation surges across a dataset of 85,033 references. Table 5 lists the top ten most co-cited references in proteomics-related obesity research, each cited at least 36 times. The most frequently co-cited work was by Cox et al. (2008), *Nature Biotechnology*, cited 84 times, followed by Huang da W et al. (2009), *Nature Protocols*, with 70 citations.

**Table 5:** Most commonly co-cited references in proteomics-obesity literature

Rank	Title	Journals	Authors	Year	Citations
1	MaxQuant enables high peptide identification rates, individualized p.p.b.-range mass accuracies and proteome-wide protein quantification	Nature Biotechnology	Cox J et al	2008	84
2	Systematic and integrative analysis of large gene lists using DAVID bioinformatics resources	Nature Protocols	Huang da W et al	2009	70
3	A rapid and sensitive method for the quantitation of microgram quantities of protein utilizing the principle of protein–dye binding	Analytical Biochemistry	Bradford MM	1976	67
4	Controlling the False Discovery Rate: A Practical and Powerful Approach to Multiple Testing	Journal of the Royal Statistical Society: Series B (Methodological)	Benjamini Y et al	1995	55
5	Universal sample preparation method for proteome analysis	Nature Methods	Wiśniewski JR et al	2009	48
6	Mass spectrometric sequencing of proteins silver-stained polyacrylamide gels	Analytical Chemistry	Shevchenko A et al	1996	40
7	The PRIDE database and related tools and resources in 2019: improving support for quantification data	Nucleic Acids Research	Perez-Riverol Y et al	2019	39
8	The Perseus computational platform for comprehensive analysis of (prote)omics data	Nature Methods	Tyanova S et al	2016	39
9	Cytoscape: a software environment for integrated models of biomolecular interaction networks	Genome Research	Shannon P et al	2003	36
10	Positional cloning of the mouse obese gene and its human homologue	Nature	Zhang Y et al	1994	36

**Fig. 6:**
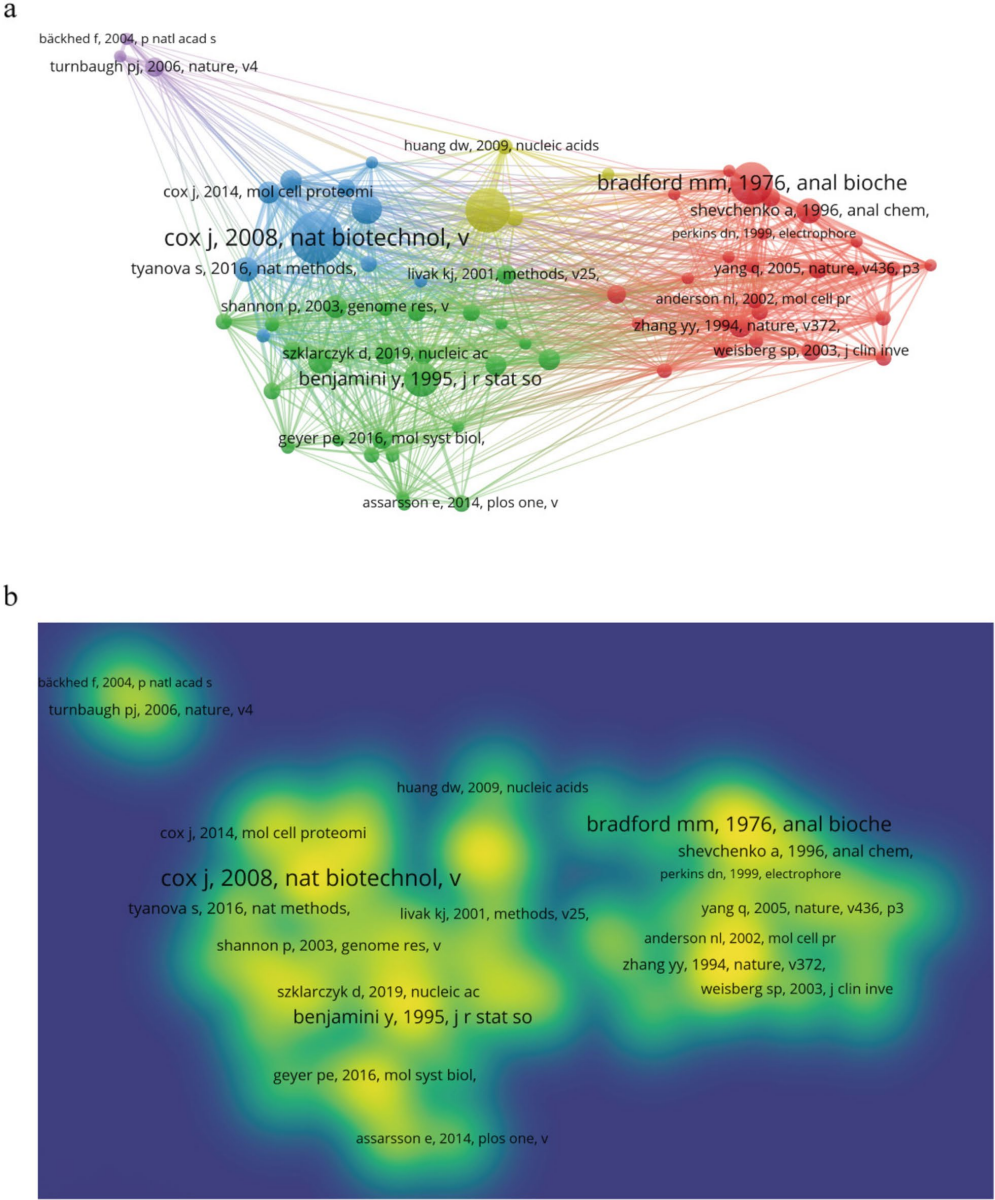
Co-citation collaboration map generated by VOSviewer. **a** Network visualization of co-cited references. **b** Density visualization of co-cited references

**Fig. 7:**
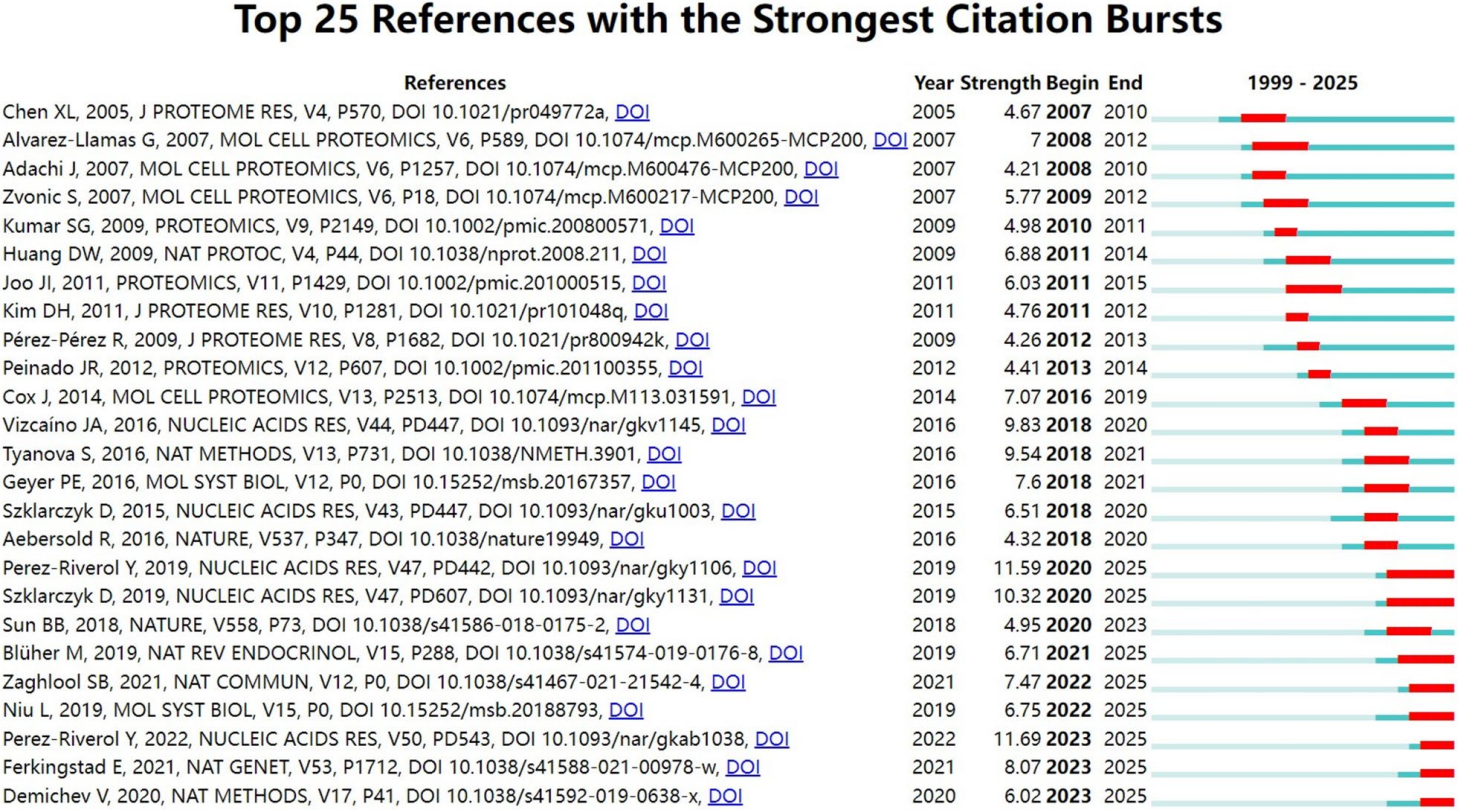
The top 25 high-cited references with the strongest citation bursts, generated by CiteSpace

The co-citation network (Fig. 6a, b) revealed close linkages among foundational works, particularly those introducing key analytical platforms like MaxQuant. In parallel, citation burst analysis (Fig. 7) identified 25 references with rapid citation increases over time. The top-ranked burst was Perez-Riverol et al. (2023–2025; strength = 11.69), followed by another work by the same author and by Szklarczyk et al. (strength = 10.32). These bursts indicate periods of heightened attention to key methodological resources and databases. Overall, burst strengths ranged from 4.21 to 11.69, with durations from 1 to 5 years.

**Fig. 8:**
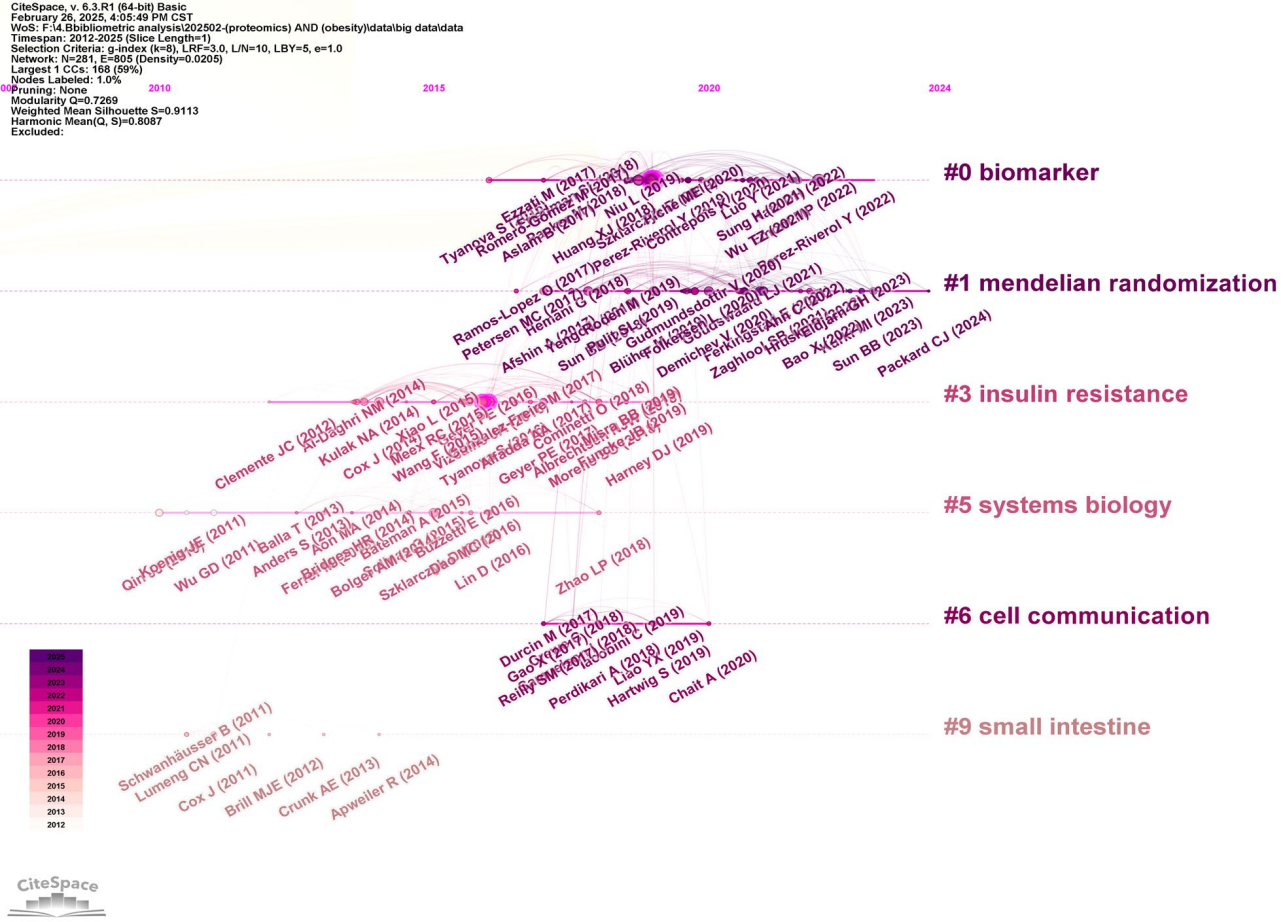
Citespace visualization map of timeline view

Figure 8 further depicts the temporal clustering of co-cited references, revealing six major themes. Early research emphasized insulin resistance, systems biology, and the small intestine, whereas recent studies have focused on biomarkers, Mendelian randomization, and intercellular communication, reflecting the field’s evolving priorities.

### Analysis of keyword co-occurrence

To complement citation-based insights, we sorted the recurring keywords and identified the top 20 words with the highest frequency (Table 6). This was conducted by combining similar keywords with the most representative topics in proteomics for obesity researches as comprehensively as possible. Given that “proteomics” and “obesity” were part of the search strategy, their high frequency is unsurprising and not inherently meaningful.

**Table 6:** Twenty most frequent author keywords across selected studies

Keyword	Occurrences	TLS	Keyword	Occurrences	TLS
Proteomics	469	866	Metabolic syndrome	45	109
Obesity	338	695	Metabolism	45	100
Proteome	87	148	Liver	38	84
Inflammation	81	175	Mitochondria	38	102
Metabolomics	81	203	Biomarkers	36	73
Insulin resistance	77	184	Type 2 diabetes	35	71
Diabetes	67	166	Transcriptomics	34	105
Mass spectrometry	62	139	Oxidative stress	31	56
Adipose tissue	57	133	NAFLD	28	62
High-fat diet	45	88	Lipid metabolism	26	48

**Fig. 9:**
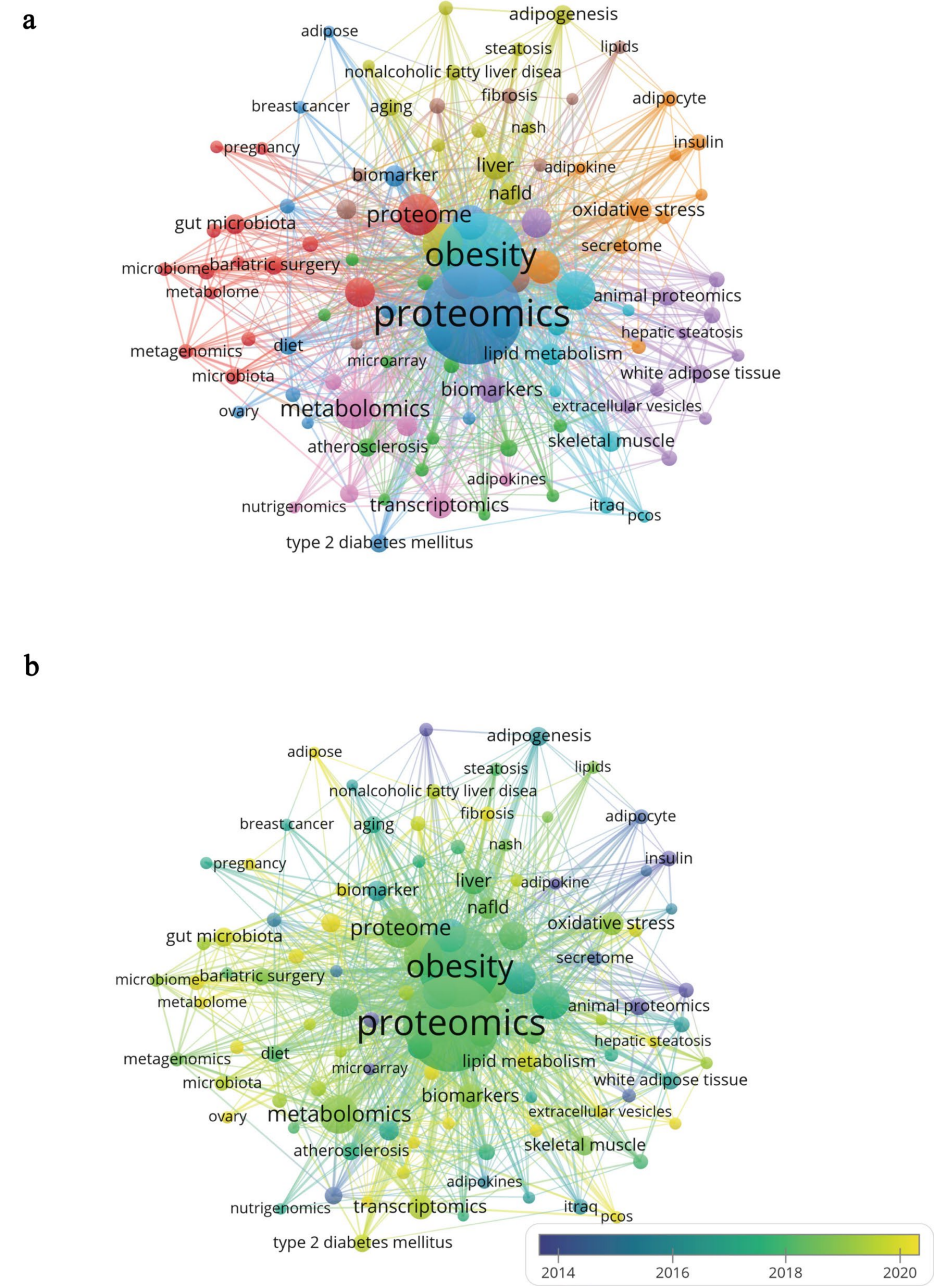
The network (**a**), overlay (**b**) of keyword co‑occurrence on proteomics for obesity researches

Keyword co-occurrence analysis was used to identify major research themes and their interconnections (Fig. 9a, b). As visualized in Fig. 9a, nine major clusters emerged. Cluster 1 (red) centers on multi-omics approaches and the gut microbiota, with terms like metagenomics, microbiome, and metaproteomics, reflecting growing interest in microbial contributions to metabolic disorders. Keywords such as bariatric surgery and weight loss highlight clinical management, while pregnancy and saliva suggest a shift toward specific populations and non-invasive sampling. Cluster 2 (green) focuses on metabolic and cardiovascular comorbidities, highlighting the frequent use of integrative omics and causal inference methods, including Mendelian randomization. Given the complexity of the full network, we highlight Cluster 1 and Cluster 2 as representative examples of distinct but prominent thematic directions. These clusters capture both mechanistic and translational aspects of obesity research. Additional clusters also contribute to the field’s diversity, though not discussed in detail.

To further explore how these research foci have changed over time, a temporal overlay analysis was conducted. As shown in Fig. 9b, it illustrates a shift from basic research on adipokines and insulin to more systemic and translational topics, including lipid metabolism, gut microbiota, type 2 diabetes, and PCOS. This trend underscores a broadening of research focus toward clinical application and precision medicine.

## Discussion

Obesity is a complex metabolic condition involving diverse biological pathways and significant inter-individual variation. Traditional metrics such as body mass index (BMI) are limited in capturing differences in fat distribution and metabolic health status [9]. Identifying biomarkers that can predict these complications at an early stage is, therefore, essential for effective clinical intervention [7]. In this context, proteomics has emerged as a valuable approach for identifying protein biomarkers associated with obesity-related complications, offering insights into disease mechanisms and opportunities for improved diagnosis and treatment planning.

### Global knowledge structure

Our bibliometric analysis reveals a steady growth in proteomics research related to obesity over the past two decades. The number of studies rose sharply after 2010, peaking in 2022. This trend may reflect both advances in analytical technologies and heightened attention to translational applications of proteomics. The slight decline observed afterward may be attributed to indexing delays in bibliographic databases and an ongoing shift in research focus toward integrative and translational directions, which are not always consistently categorized under traditional proteomics labels. The sustained citation growth since 2010 suggests that the field has gained stable academic recognition.

The field is shaped by strong international participation, led by the United States, China, and Germany. Active bilateral collaboration, particularly between the U.S. and China, plays a major role in driving scientific output. Prominent institutions such as the University of Copenhagen, Maastricht University, and Harvard Medical School have served as research hubs, often coordinating multicenter efforts. These collaborative structures reflect the global scope of proteomics research in obesity and underscore the value of sustained institutional networks.

Several scholars have made substantial contributions to the field. Notably, Yun Jong Won and Choi Jung Won have maintained high publication activity, while co-citation analysis highlights influential figures such as Cox J and Hotamisligil GS. Their foundational work on proteomic tools and metabolic mechanisms has shaped both methodological standards and scientific direction.

### Global research priorities and trends

Our keyword and reference analyses demonstrate a clear thematic shift over time. Early studies focused on insulin signaling, adipokines, and animal models, reflecting efforts to define basic molecular processes. More recent attention has turned to clinically relevant areas such as lipid metabolism, gut microbiota, type 2 diabetes, and polycystic ovary syndrome (PCOS). The increasing use of multi-omics and causal inference methods, including The Mendelian randomization, reflects a growing emphasis on translational applications. This analysis draws on the average year in which each keyword appeared, as shown in the overlay map. Although this measure does not reflect the most recent research activity, it is commonly used in bibliometric studies to indicate when certain topics began to attract broader and sustained attention. The observed time pattern reflects a gradual shift in research focus, moving from cellular and molecular mechanisms to more integrated investigations of systemic metabolism and related diseases. The overlay visualization helps demonstrate how the field has expanded toward more complex and clinically relevant directions.

Among the emerging themes, lipid metabolism has gained prominence due to its central role in fat accumulation and metabolic regulation [10]. Glucagon-like peptide-1 (GLP-1), for instance, has emerged as a potential therapeutic target due to its regulatory effects on lipid processing and fat distribution [11]. Likewise, the gut microbiota has been linked to chronic inflammation in obesity, shifting attention toward host-microbe interactions [12–14]. Type 2 diabetes and PCOS remain critical complications, driving interest in early biomarkers and personalized management strategies [15, 16]. Weight loss and antidiabetic therapies have shown effectiveness in improving metabolic outcomes and supporting diabetes remission [17, 18]. Evidence from both observational study and randomized controlled trials indicates that bariatric surgery leads to significant improvements in metabolic and reproductive outcomes among women with obesity and PCOS [19, 20]. These biological themes reflect both the complexity of obesity and the areas where proteomics research has become increasingly active. The proteomic analyses have helped identify key protein markers associated with inflammation, glucose control, and lipid dysregulation [21–23]. While these findings highlight the clinical relevance of proteomics, our study emphasizes how this field has evolved in terms of research output and thematic direction, rather than focusing on specific molecular pathways.

In summary, this bibliometric study outlines the growth trajectory and evolving priorities of proteomics research in obesity. By mapping publication trends, author influence, institutional collaboration, and research hotspots, our analysis provides a foundation for understanding how the field has developed and where future opportunities may lie. These findings may support strategic planning, interdisciplinary collaboration, and the identification of underexplored topics for further investigation.

### Limitations

This study was based on data from the WoSCC, a widely used and trusted source for bibliometric research. Although other databases like Scopus and PubMed may include additional publications, WoSCC is well-recognized for its indexing quality and compatibility with bibliometric tools. We believe the data used here offer a solid reflection of global research trends in proteomics and obesity. Future work may benefit from cross-database integration to further enhance coverage and robustness.

## Conclusion

This study presents a comprehensive bibliometric analysis of proteomics research in obesity based on publications from the Web of Science Core Collection between 1999 and 2025. The steady increase in publication output, especially since 2018, reflects growing interest in the field and the adoption of advanced technologies such as mass spectrometry. This analysis outlines the current research landscape, highlights key contributors and emerging topics, and provides a foundation for guiding future research directions and promoting international collaboration.

## Data Availability

No datasets were generated or analyzed during the current study.
